# SnoN Stabilizes the SMAD3/SMAD4 Protein Complex

**DOI:** 10.1038/srep46370

**Published:** 2017-04-11

**Authors:** Karin Walldén, Tomas Nyman, B. Martin Hällberg

**Affiliations:** 1 Department of Cell and Molecular Biology, Karolinska Institutet, 171 77 Stockholm, Sweden; 2 Department of Medical Biochemistry and Biophysics, Karolinska Institutet, 171 77 Stockholm, Sweden; 3 Röntgen-Ångström-Cluster, Karolinska Institutet Outstation, Centre for Structural Systems Biology, DESY-Campus, 22603 Hamburg, Germany; 4 European Molecular Biology Laboratory, Hamburg Unit, 22603 Hamburg, Germany

## Abstract

TGF-β signaling regulates cellular processes such as proliferation, differentiation and apoptosis through activation of SMAD transcription factors that are in turn modulated by members of the Ski-SnoN family. In this process, Ski has been shown to negatively modulate TGF-β signaling by disrupting active R-SMAD/Co-SMAD heteromers. Here, we show that the related regulator SnoN forms a stable complex with the R-SMAD (SMAD3) and the Co-SMAD (SMAD4). To rationalize this stabilization at the molecular level, we determined the crystal structure of a complex between the SAND domain of SnoN and the MH2-domain of SMAD4. This structure shows a binding mode that is compatible with simultaneous coordination of R-SMADs. Our results show that SnoN, and SMAD heteromers can form a joint structural core for the binding of other transcription modulators. The results are of fundamental importance for our understanding of the molecular mechanisms behind the modulation of TGF-β signaling.

The TGF-β family of extracellular growth factors regulates a diverse spectrum of cellular processes including cell proliferation, adhesion, migration, cell-fate determination and apoptosis. Defects in the signaling downstream of TGF-β are associated with metastatic cancer. TGF-β signaling converges to a set of intracellular SMAD proteins that further transfers the signal to changes in transcription. Receptor-regulated SMAD proteins (R-SMADs) are activated by phosphorylation of C-terminally located serine residues. This phosphorylation enables a heterocomplex formation between R-SMADs and the common-mediator SMAD (Co-SMAD; SMAD4). The heterocomplex is imported into the nucleus where it activates or represses transcription at specific SMAD-binding sequences at promoter sites^1^,^2^. Several co-crystal structures of different SMADs bound to target DNA have been determined^3^,^4^,^5^. In addition to binding DNA, SMADs often function via association with other transcription regulators, such as histone acetyltransferase co-activators p300 and CBP^6^,^7^, histone deacetylase (HDAC) co-repressors^8^ and intracellular parts of the Delta-like protein which may play an important role in bi-directional Notch-Delta signaling^9^.

Furthermore, there are direct modulators of TGF-β signaling and the most studied of these modulators are Ski and SnoN, which although sharing 37% sequence identity, have individual essential roles in embryo development and the differentiation of adult stem cells. Levels of both proteins are tightly regulated by proteosomal degradation and *via* SMAD transcriptional activation^10^,^11^. The SnoN-SMAD4 complex negatively regulates SnoN transcription at basal SnoN levels effectively leading to negative feedback regulation of SnoN levels^12^. Both Ski and SnoN are well-known oncoproteins whose expression is elevated in several tumor cell lines resistant to the TGF-β repression of cell growth^13^,^14^,^15^,^16^,^17^,^18^. Furthermore, Ski and SnoN have anti-oncogenic behavior in some cancer cells^19^,^20^. For SnoN, this may be explained by a dual role in TGF-β signaling in which low levels of SnoN promote TGF-β dependent transcription while high levels antagonize it^21^.

SnoN and Ski bind to promoter-bound SMAD transcription factors such as the R-SMAD/Co-SMAD heteromer, which in turn recruits co-repressors such as HDAC, NCoR1 or Sin3A to the promoter site^22^,^23^,^24^,^25^,^26^,^27^. SnoN and Ski also compete for the binding of SMADs with the co-activators p300 and CBP. Interestingly, Ski has been shown to disrupt the R-SMAD/Co-SMAD heteromer^28^, while no such data is available for SnoN.

Ski and SnoN have similar domain architectures featuring an unstructured N-terminal region with an R-SMAD binding site; a Dachshund-homology domain and a SMAD4-binding SAND domain; and, at their end, an extended, unstructured C-terminal tail^28^,^29^. Crystal structures have been determined of the Dachshund-homology domain of SnoN^30^. Furthermore, a crystal structure of the SAND domain of Ski, in complex with the MAD homology 2 (MH2) domain of SMAD4, is available. This latter structure, in combination with accompanying biochemistry work, suggested a model in which Ski disrupts the active SMAD heterotrimer and thereby inhibits SMAD transcriptional regulation^28^. Based on their different signaling roles, we hypothesized that Ski and SnoN may act differently on R-SMAD/Co-SMAD heteromers i.e. such that Ski may destabilize and SnoN may stabilize R-SMAD/Co-SMAD complexes. To this end, we studied the effect of SnoN on the heteromeric complex of SMAD3 and SMAD4 through a range of biochemical and biophysical techniques.

## Results and Discussion

### SMAD3, SMAD4 and SnoN Form a Stable Complex

**Figure 1:**
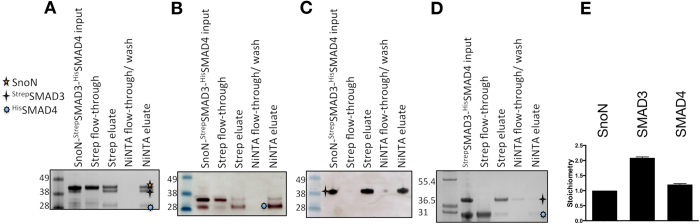
Analysis of SnoN/SMAD Interactions. Affinity purification of a complex including truncated forms of untagged SnoN (M1-S356, 38.6 kDa), Strep II tagged SMAD3 (MH2 domain (S423E, S425D), 33.6 kDa) and His tagged SMAD4 (MH2 domain, 28.3 kDa) (**A**–**C**). From left, SnoN-^Strep^SMAD3-^His^SMAD4 input; sample not binding to the Strep-Tactin column (Strep flow-through); Strep-Tactin eluate (Strep eluate); Strep-Tactin elute was loaded directly onto the Ni-NTA column; any proteins not binding to the Ni-NTA column (Ni-NTA flow-through/wash); and Ni-NTA eluate containing the isolated complex of SMAD3, SMAD4 and SnoN. Integrity and identity of SMAD4 was confirmed by MALDI-MS/MS mass spectrometry analysis of trypsinated protein extracted from the gel (Supplemental information). (**A**) Coomassie stained gel. (**B**) Western blot analysis using anti-hexahistidine tag antibody recognizing ^His^SMAD4. The lowest band corresponds to SMAD4, as confirmed by mass spectrometry. (**C**) Western-blot analysis using anti-Strep II tag antibody recognizing ^Strep^SMAD3. (**D**) Affinity purification as in (**A**–**C**) but excluding SnoN. (**E**) Stoichiometry of SnoN-^Strep^SMAD3-^His^SMAD4 complex assessed by fluorescence labeling of cysteines in the NiNTA eluate seen in A by a maleimide derivative of IRDye 800CW (LI-COR Biotechnology GmbH). Error bars are for the standard deviation of three individual measurements, see Supplementary information. SeeBlue^®^ Plus2 Pre-stained Protein Standard (Invitrogen) and Mark12 unstained standard (Thermo Fisher Scientific) were used for (**A**–**C** and **D**), respectively, with relevant molecular weights indicated to the left of each gel. IgG antibody conjugated to alkaline phosphatase was used for blots in (**B** and **C**). Please see the experimental procedures section for further details.

In order to study the interaction between SnoN and SMAD3/SMAD4 biochemically, untagged SnoN (M1-S356) – containing both SMAD3-binding regions and the SMAD4-binding SAND domain^31^ (Figure S1) – was mixed with the purified Strep II-tagged MH2 domain of SMAD3 (S423E, S425D) (^Strep^SMAD3) and the His-tagged MH2 domain of SMAD4 (^His^SMAD4). After complex formation, the mixture was run over two columns with selectivity for either SMAD3 or SMAD4. Unexpectedly, ^Strep^SMAD3, ^His^SMAD4 and SnoN formed a stable complex (Fig. 1A–C and Figure S2). When repeating this affinity purification procedure with mixed ^Strep^SMAD3 and ^His^SMAD4 but without SnoN, the ^Strep^SMAD3/^His^SMAD4 formed a less stable complex than the SnoN/^Strep^SMAD3/^His^SMAD4, indicating that SnoN stabilizes the SMAD3/SMAD4 complex (compare 1A and 1D). To further assess the stoichiometry of the SnoN-SMAD3-SMAD4 complex, we used near-infrared fluorescence imaging with a cysteine-specific dye. The resulting stoichiometry was 1.0:2.1:1.2 (SnoN:SMAD3:SMAD4) (Fig. 1E and Figure S3). This supports that SnoN forms a complex with an active SMAD3-SMAD4 heterotrimer. This result differs significantly from the behavior of Ski, where the SAND domain of Ski disrupted the R-SMAD/Co-SMAD complexes^28^.

### Crystal Structure Reveals Two Distinct SnoN/SMAD4 Forms

**Figure 2:**
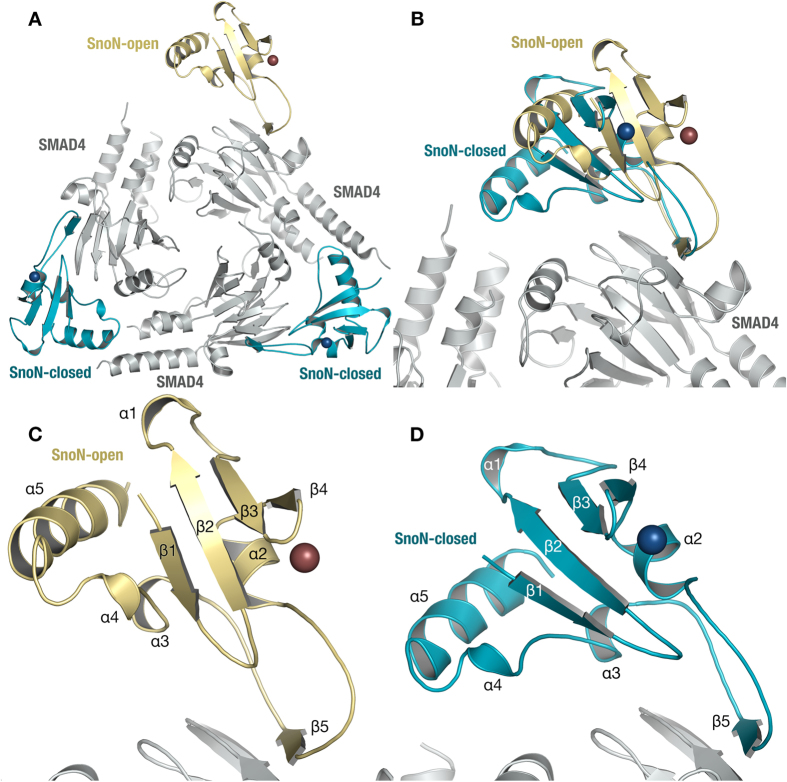
Overall Structure of SnoN-SMAD4. (**A**) Content of asymmetric unit, with SnoN-SMAD4 as trimers of heterodimers, with SMAD4 (grey) mediating most of the trimer contacts, one open (beige) and two SnoN molecules in closed conformation (cyan). Bound Zn ions are indicated in brown and blue for open and closed conformation SnoN, respectively. (**B**) Open and closed conformations of SnoN with the interacting SMAD4s superimposed. (**C**) Close-up of open-conformation SnoN. (**D**) Close-up of closed-conformation SnoN. Secondary structure elements are indicated as α-helix (α) and β-strand (β) with numbers increasing from the N-terminal to the C-terminal. Color codes as in (**A**).

Earlier studies have shown that the interaction between Ski and SMAD4 is critical for Ski’s disruption of the R-SMAD/SMAD4 complex^28^. Therefore, to rationalize the SnoN complex-stabilization results described above at a molecular-mechanistic level, we crystallized a complex between the SAND domain of SnoN (T238-S356) and the MH2 domain of SMAD4 (I314-Q549). The complex crystallizes as trimers of SnoN-SMAD4 heterodimers (Fig. 2A), where SnoN interacts with SMAD4 in either an “open” or a “closed” binding mode (Fig. 2B–D). A striking structural feature is the strand complementation observed between SnoN β-strand β5 and the edge strand of the SMAD4 MH2 central β-sheet (Fig. 2C,D). Similar interactions are common at the stable protein-protein interfaces of obligatory dimers such as mitochondrial poly-A polymerases^32^.

### Interactions Bridging SnoN and SMAD4, Seen in Both Open and Closed Forms, are Specific and Evolutionary Conserved

**Figure 3:**
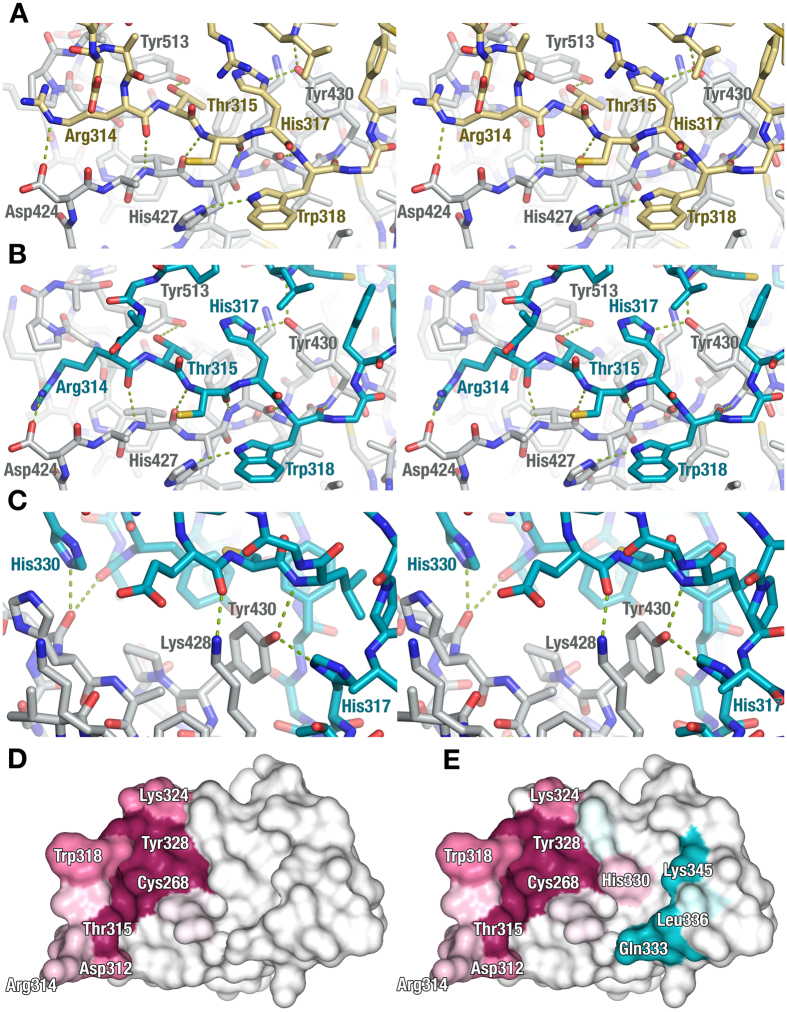
Interactions Between SnoN and SMAD4. I(**A**) Open conformation interactions between SnoN (beige) and SMAD4 (grey). (**B**) Closed conformation interactions between SnoN (cyan) and SMAD4 (grey). (**C**) Additional interactions between SnoN (cyan) and SMAD4 (grey), which are part of SnoN-SMAD4 interactions in the closed conformations only. (**D**) Open conformation of SnoN. Residues involved in SMAD4 interactions in SnoN, according to the buried surface model as implemented in PISA^42^, are colored according to level of evolutionary conservation, going from fully conserved (violet-red) to no conservation (blue-green), calculated using the Consurf server (http://consurf.tau.ac.il/)^43^. (**E**) ‘Closed’ conformation of SnoN. Same coloring as in (**D**).

When comparing the open and closed SnoN-binding modes, we find that all SnoN-SMAD4 interactions that are present in the open conformation (Fig. 3A) are also present in the closed state (Fig. 3B). These interactions include parallel β-backbone hydrogen bonds between the conserved strand β5 and adjacent loop regions (residue Arg314-Trp318) in SnoN and the edge β-strand (residues 424–430) of SMAD4. The interactions also include binding of SnoN Trp318 in a hydrophobic cleft in SMAD4 lined by Leu414, Ile429, Ile435 and His427. To further strengthen the SnoN-SMAD4 heterodimer interface, His427 offers a side-chain hydrogen bond to Trp318, and Arg314 in SnoN forms an ionic interaction with Asp424 in SMAD4. Beyond these common consensus interactions, the closed state contains additional hydrogen bonds between SnoN (residues Lys313, Lys324 and His330) and SMAD4 (Fig. 3C). These additional hydrogen bonds appear to have suboptimal geometry and distances, which makes their precise contribution to interface stabilization questionable.

The open SnoN-SMAD4 state reveals a novel binding mode whereas the relative orientation of SnoN and SMAD4 in the closed conformation resembles that of the previously reported Ski-SMAD4 structure (PDB code 1MR1)^28^. Ski and SnoN share 37% overall amino-acid sequence identity, and 43% sequence identity for the SAND domain (Figure S4), and despite significant sequence similarity, Ski and SnoN participate in different essential cellular processes^10^,^11^. A comparison of our SnoN-SMAD4 structure with that of Ski-SMAD4 (PDB code 1MR1) shows that the consensus interactions common to the closed and open states in our SnoN-SMAD4 structure are strongly conserved between SnoN-SMAD4 and Ski-SMAD4, particularly the β5 interactions. Interestingly, the additional interactions of the closed SnoN-SMAD4 state are not conserved between SnoN and Ski (Figure S4). Furthermore, mapping the evolutionary conservation to the surface of SnoN highlights that SnoN residues involved in consensus interactions (present in both open and closed states) are highly conserved, while those unique to the closed conformation are more diverse (Fig. 3D–E). This may indicate that the explanation for the difference between Ski’s and SnoN’s interaction behaviour with the co-SMAD/R-SMAD complex lies in the non-conserved sequence regions involved specifically in the closed state.

It is evident that the observed binding modes of SnoN and Ski to SMAD4 may be affected by crystal packing or non-crystallographic symmetry. In the SnoN-SMAD crystal structure presented here, SnoN-SMAD4 crystallizes as a trimer of heterodimers (Figure S5). Furthermore, the SnoN protomers in the closed conformation packs against other SnoN protomers in the closed conformation while the single SnoN protomer in the open conformation packs against another SnoN protomer also in the open conformation. This way the angle relative to SMAD4 is restricted – but not as restricted as the angles for the two SnoN protomers in the closed state. In the Ski-SMAD4 case, the asymmetric unit is built up with two SMAD4 protomers and two Ski protomers where the Ski protomers interact with each other and packs crystallographically with the C-terminal part of SMAD4^28^. Hence, it is conceivable that the crystallographic packing enforces the closed state observed in the crystal structures of both the SnoN and Ski SMAD4 co-crystal structures. Therefore, it is possible that Ski may also bind SMAD4 in two conformations in solution, where the existing Ski-SMAD4 crystal structure^28^ may have captured only the closed state. However, it is outside the scope of this work to investigate this possibility for Ski further.

### Kinetics Indicate Existence of One Major SnoN/SMAD4 Binding Mode in Solution

**Figure 4:**
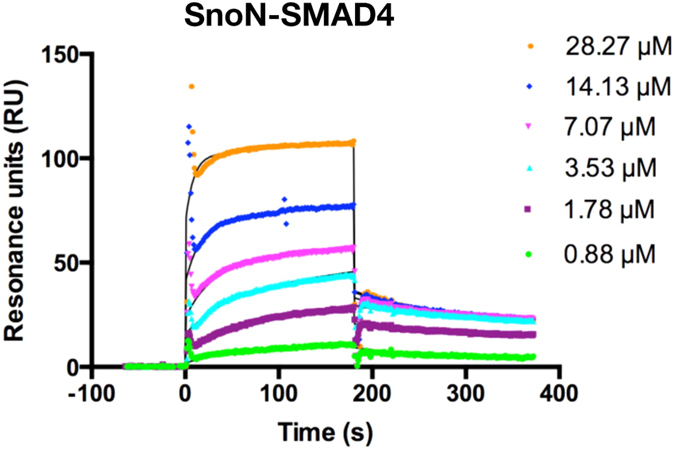
Surface plasmon resonance-based analysis of SMAD4-binding to SnoN. Corrected response of various concentrations of SnoN on SMAD4. Sensorgrams with curve fittings (black continuous line) for each titration experiment are shown. SnoN concentrations used for each titration/sensorgram are indicated.

We used surface plasmon resonance (SPR) techniques to test whether there are several binding modes for SnoN/SMAD4 also in solution. To this end, we titrated SnoN onto a SPR chip coated with SMAD4. The resulting sensorgrams could be easily fitted to a simple one-step binding model (Fig. 4). From this, the association and dissociation rate constants for the complex formation, *k*_a_ and *k*_d_ were determined to be 1.2*10^5^ M^−1^s^−1^ (SE = 3.0*10^3^ M^−1^s^−1^) and 0.129 s^−1^ (SE = 1.1*10^−3^ s^−1^), respectively. The *k*_a_ and *k*_d_ in turn gave the equilibrium dissociation constant *K*_D_ = *k*_d_/*k*_a_ = 1.1 μM for the SnoN-SMAD4 interaction. These results show that SnoN and SMAD4 interact through one dominating binding mode and the level of affinity is similar to what is typical in other signaling complexes^33^. Thus, based on these structural, evolutionary and biochemical considerations, we suggest that the open SnoN-SMAD4 conformation represents the biologically and functionally more relevant binding mode.

### Structural Analysis Indicates how SnoN can Recognize SMAD Heterotrimers without Intermolecular Clashes

**Figure 5:**
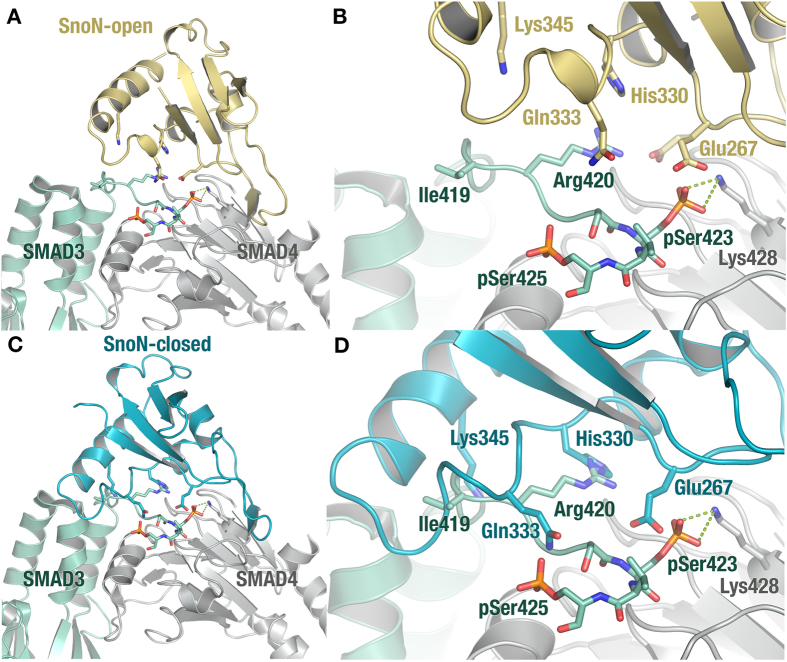
SnoN-SMAD4 Superimposed on the SMAD3-SMAD4 Heterotrimer Structure. (**A**) Open conformation of SnoN (beige) superimposed based on the interacting SMAD4 with the structure of SMAD3-SMAD4 (green-grey) (PDB code 1U7F^34^) (**B**) Close-up of (**A**). Glu267 is shown in both the conformation found in our complex structure and in its most preferred rotamer (50% transparent), which is compatible with the binding of an activated SMAD3-SMAD4 heteromer. (**C**) Closed conformation of SnoN (cyan) superimposed based on the interacting SMAD4 in the structure of SMAD3-SMAD4 (green-grey). (**D**) Close-up of (**C**). Phosphoserine residues of SMAD3 indicated as pSer423 and pSer425, respectively.

TGF-β-activated (phosphorylated) SMADs assemble into heterotrimers consisting of two phosphorylated R-SMAD molecules and one SMAD4 molecule. The crystal structures of the heterotrimers of the SMAD2/SMAD4 and SMAD3/SMAD4 MH2 domains have been determined^34^. The previous superposition of the structures of Ski-SMAD4 and a SMAD2 homotrimer revealed potential intermolecular clashes upon Ski-SMAD association, which suggested a possible molecular mechanism for the inhibition of TGF-β signaling *via* disruption of the R-SMAD/Co-SMAD heterotrimer^28^. In contrast, the open conformation of our SnoN-SMAD4 structure can be superposed on the SMAD3-SMAD4 heterotrimer without significant steric hindrance between SMAD3 and SnoN (Fig. 5A). While the space occupied by Glu267 coincides with that of the phosphorylated Ser423 (pSer423) in the SMAD3-SMAD4 structure, the Glu267 side chain could easily adjust to accommodate pSer423. Hence, from a structural viewpoint, SnoN would be fully capable of recognizing SMAD3-SMAD4 without disrupting the heterotrimer, which suggests a different mechanism for SnoN than that suggested for Ski^28^.

In contrast, when overlaying the structures of the closed SnoN-SMAD4 state and SMAD3-SMAD4 (Fig. 5B), we note significant overlap between SnoN and SMAD3. These sterically incompatible regions are all located outside the SnoN-SMAD4 and SMAD3-SMAD4 interfaces, and can therefore not be directly implied in the R-SMAD/Co-SMAD interaction. Importantly, no SnoN-interacting SMAD4 residue overlaps/clashes with a SMAD3-SMAD4 interaction, and in fact, only Lys428 of SMAD4 interacts with both SnoN and SMAD3. Additionally, the crystal packing of the SnoN-SMAD4 molecules as trimers of heterodimers strongly supports the notion that a SMAD trimer is fully compatible with bound SnoN (Figure S5).

## Conclusions

We have successfully isolated a stable SMAD3/SMAD4/SnoN complex, and have shown that this complex is more stable than the SMAD3/SMAD4 complex in the absence of SnoN. Our kinetic analysis confirms the high affinity of the SnoN SAND domain for SMAD4, and that association follows a one-step binding model. This suggests that only one form, the open state, of SnoN-SMAD4 is present in solution.

The structure of the open conformation of SnoN-SMAD4 provides the basis for a mechanism for SnoN-mediated modulation of TGF-β signaling, which is distinct from that previously suggested based on the Ski-SMAD4 structure^28^. In the open form, SnoN would be able to bind an intact SMAD heterotrimer without intermolecular clashes or further structural readjustment. For the closed state, however, structural reorganization within the SMAD heterotrimer, similar to that seen for the Ski-SMAD4 complex^28^, would be required to enable SnoN binding. Hence, Ski and SnoN may use two distinct structural mechanisms for modulating SMAD signaling.

The structure of our open SnoN-SMAD4 state suggests that SnoN can bind to an active SMAD heterotrimer to offer a stable scaffold for binding of co-repressors or co-activators, whereas the conformational state associated with Ski binding could result in inactivation of SMAD signaling *via* disruption of the SMAD heterotrimer^28^. Importantly, this active SnoN-SMAD model is compatible with expansion into larger functional complexes where additional co-repressors/co-activators are included during transcriptional regulation. Interestingly, our biochemical and structural data indicates a different mode of modulation of SnoN compared to that of Ski, which may reflect differences in the biological function of these modulators in TGF-β signaling – an insight that opens up new avenues for modulator-specific therapeutic intervention in their respective roles in metastatic cancers.

## Methods

### Cloning, expression and purification of recombinant proteins

The SAND domain of SnoN (T238-S356), SnoN (M1-S356) and the MH2 domain of SMAD4 (I314-Q549) were each cloned into the pET-derived vector pNIC28Bsa4, providing a N-terminal hexahistidine tag with integrated TEV site (MHHHHHHSSGVDLGTENLYFQ*SM). For the SPR analysis, SnoN (T238-S356) was cloned into the pET-derived vector pNicBio3 that provides a biotinylation sequence in the C-terminus. SMAD3 (E145-D425, including mutations S423E and S425D to mimick the phosphoserines in the activated SMAD3^35^) was cloned into the vector pASKIBA13 + (IBA) providing a N-terminal Strep II tag with integrated thrombin cleavage site. Each construct was expressed in *E. coli* strain KRX including the pRARE2 plasmid (for expression of rare tRNAs). Each construct was expressed at 18 °C using standard methods for growth and expression, with 0.5 mM isopropyl-β-D-thiogalactopyranoside and 0.2% rhamnose (SnoN/SMAD4) or 200 ng/mL anhydrotetracyclin (SMAD3) in terrific broth supplemented with 8 g/L glycerol (SMAD4 and SMAD3) or Luria-Bertani broth (SnoN). Harvested cells were resuspended in lysis buffer (50 mM HEPES, pH 8; 300 mM NaCl; 10% glycerol; 0.5 mM PMSF and 0.5 mM tris(2-carboxyethyl)phosphine (TCEP)), and cells were disrupted by high-pressure homogenization followed by centrifugation for 20 min at 40,000 g.

Proteins were purified with Ni-affinity (SMAD4 and SnoN constructs) or Strep II-affinity (SMAD3) chromatography and gel filtration chromatography, using fast protein liquid chromatography ÄKTA instruments (GE Healthcare). 5-ml Ni-charged HisTrap HP columns (GE Healthcare), 5-mL StrepTrap HP columns (GE Healthcare) and either a HiLoad 16/60 Superdex 200 (SMAD4), HighPrep 16/60 Sephacryl 300 (SMAD3) or a Superdex 200 HR10/30 (SnoN constructs) gel filtration column (GE Healthcare) were used. HisTrap HP columns were equilibrated in buffer A (20 mM HEPES, pH 8.0; 300 mM NaCl; 10% glycerol; and 2 mM TCEP), washed in Buffer A supplemented with 30 mM imidazole, and His-tagged proteins were eluted using a gradient of buffer A and buffer B (buffer A containing 500 mM imidazole). His tags of SnoN constructs were cleaved off using TEV protease at a molar ratio of 1/30, TEV/SnoN, during dialysis into buffer A, overnight at 4 °C. SnoN protein without affinity tags was separated from remaining tagged protein and TEV protease, by a second Ni affinity purification step, then with the desired protein without affinity tag in the flow-through. StrepTrap columns were equilibrated in buffer A and Strep II tagged protein was eluted with buffer A supplemented with 2.5 mM desthiobiotin, according to manufacturer’s protocol. Gel-filtration columns were equilibrated in buffer A. The retention volumes of SMAD4, SMAD3 and SnoN corresponded to that of a monomer, trimer and a monomer/dimer, respectively. The SnoN-SMAD4 complex was reconstituted at a molar ratio of 1:1, for 2 h on ice, then run on a Superdex 200 HR10/30 column, eluting with a retention volume corresponding to a heterodimer. After purification, protein identities were confirmed by mass spectrometry (high performance liquid chromatography-electrospray ionization-mass spectrometry).

### Isolation of a Complex Between SMAD3, SMAD4 and SnoN

StrepII-tagged SMAD3 (^Strep^SMAD3), hexahistidine-tagged SMAD4 (^His^SMAD4) and untagged SnoN (M1-S356) were purified to high purity as described above. The proteins were mixed at a molar ratio of SnoN:SMAD3:SMAD4 of 2.5:1:1, then incubated for 2 h on ice, followed by two affinity purification steps: First using 1 mL Strep-Tactin resin (high capacity: IBA) in 20-mL gravity flow columns (BioRad) and then a 100 μL Ni-NTA resin (Thermo Scientific) in 1.5 mL 0.22 μM filter spin columns (Millipore). Input samples were loaded on Strep-Tactin columns and incubated for 15 min. The incubation was followed by the collection of flow-through with 2 column volumes (CVs), a 5 CV wash step, and a 2 CV elution step. Volumes of 1 mL per eluted sample were directly added to Ni-NTA resin in 1.5 mL eppendorf tubes and incubated for 30 min, then resin was transferred to 1.5 mL spin columns and flow through was collected. This was followed by a 5 CV wash step, followed by elution using 3 CVs. Equilibration and wash steps of Strep-Tactin columns were performed using buffer A and elution step of Strep-Tactin columns was performed using buffer A supplemented with 2.5 mM desthiobiotin (Sigma-Aldrich). Ni-NTA resin was equilibrated in buffer A supplemented with 20 mM imidazol, followed by the wash step with buffer A supplemented with 50 mM imidazol and the elution step with buffer A supplemented with 500 mM imidazol. Fractions were analyzed with 4–12% Bis-Tris SDS-PAGE gel electrophoresis followed by Coomassie staining and Western blotting. Either Mouse Strep II tag antibody (Qiagen) or mouse monoclonal PentaHis antibody (Qiagen) was used followed by addition of anti-mouse IgG antibody conjugated to alkaline phosphatase (A3562, Sigma) and detection using Sigmafast BCIP/NBT AP substrate (Sigma-Aldrich).

The stoichiometry determination was carried out using fluorescence labelling of cysteines with IRDye 800CW Maleimide (LI-COR Biotechnology GmbH). The isolated SnoN-SMAD3-SMAD4 complex from the experiment described above was boiled with 1% SDS, 10 min at 98 degrees Celsius. Then it was supplemented with 50 mM Hepes, pH 7.0; 5 mM TCEP and 0.4 mM IRDye 800CW Maleimide, followed by incubation in dark for 2.5 hours. Samples were TCA precipitated and re-suspended in 4 M urea, followed by 4–12% Bis-Tris SDS-PAGE gel electrophoresis and gel scanning using the Odyssey Infrared Imaging System (LI-COR Biotechnology GmbH). Analysis was performed using Image Studio Lite (LI-COR Biotechnology GmbH). Plots and analysis was made using GraphPad Prism version 6.

### Crystallization, data collection, structure determination and refinement

Protein concentration used for crystallization was 11 mg/mL, calculated using absorbance at 280 nm and the combined extinction coefficients of SMAD4 and SnoN. The sitting drop vapor-diffusion method was used to produce SnoN-SMAD4 crystals by making drops of 2:1 protein:reservoir solution. Initial crystals were obtained in 300 nL drops, from which crystal seeds were transferred using streak seeding into 1.2 μL drops with a two-fold dilution of the protein component. Crystals were grown at 20 °C for 2 weeks with a reservoir solution consisting of 2.8–3.3 M NaCl and 0.1 M Bis-Tris, pH5.5. The crystal used for data collection was 0.1 × 0.1 × 0.2 mm in size. The crystal was flash-frozen in liquid nitrogen without further cryo protection. The crystallographic data collection was performed at beam line I04–1 at Diamond Light Source (United Kingdom). The data was processed using XDS^36^ followed by POINTLESS and SCALA^37^. Phases were obtained by molecular replacement using PHASER^38^, with the Ski-SMAD4 structure as template (PDB code 1MR1^28^). Anisotropy B-factor correction was performed using the Diffraction Anisotropy Server (http://services.mbi.ucla.edu/anisoscale/). Refinement using PHENIX 1.8.4^39^ was performed with a twinning fraction of 0.19 and the twinning operator −1/2*h-3/2*k,1/2*h-1/2*k,l. Refinement was continued with REFMAC5^40^. Model building was carried out using COOT^41^. See Table 1 for data processing and refinement statistics.

### Surface plasmon resonance based experiments

Binding experiments were performed using a BIAcore 2000 instrument (GE Healthcare). A carboxymethylated dextran chip (CM5, GE Healthcare) was used to immobilize SMAD4 (I314-Q549). A net positive charge is required for direct amine coupling; therefore, SMAD4 was diluted to a final concentration of 10 μg/mL in 10 mM sodium acetate buffer, pH 5.5. The flow cell was activated by a 1:1 solution of 100 mM N-hydroxysuccinimide and 400 mM N-ethyl-N’-(dimethyl- aminopropyl)carbodiimide. The protein was injected at 5 μL/min until approximately a 1300 response unit level of immobilized SMAD4 was reached, and then the flow cell was deactivated with 1 M ethanolamine HCl (pH 8.5). A control flow cell without immobilized ligand was activated and deactivated as indicated above. After immobilization, the channels were washed extensively with degassed sample buffer (10 mM HEPES, pH 7.4; 150 mM NaCl; 0.005% TWEEN-20; 0.5 mM TCEP) and regeneration solution (2 M NaCl). All experiments were carried out at room temperature and with a flow rate of 30 μL/min. The SnoN SAND-domain (T238-S356) at concentrations of 0–500 nM was injected for 2 min followed by a sample buffer wash for 10 min. After each cycle, the chip was regenerated with regeneration solution for 2 times 30 sec. Blank-corrected binding profiles (sensorgrams) for SnoN (T238-S356) were obtained by subtracting the response in the reference channel from the response in the binding channel. Data modification including scale transformation and background subtraction and calculations were performed with the program BIAevaluation (GE Healthcare).

## Additional Information

**Accession codes:** The crystal structure of SnoN in complex with SMAD4 have been deposited in the wwPDB under the accession code 5C4V.

**How to cite this article**: Walldén, K. *et al*. SnoN Stabilizes the SMAD3/SMAD4 Protein Complex. *Sci. Rep.* **7**, 46370; doi: 10.1038/srep46370 (2017).

**Publisher's note:** Springer Nature remains neutral with regard to jurisdictional claims in published maps and institutional affiliations.

## Supplementary Material

Supplementary Information

## Figures and Tables

**Table 1:** Data collection and refinement statistics.

Data collection	
Resolution (Å)	40.3–2.6 (2.74–2.60)*
Space group	C2
Unit cell (Å; a,b,c,β)	213.54 122.83 51.57 90.72
No. of observed reflections	110677 (14588)
No. of unique reflections	39718 (5678)
Completeness (%)	97.2 (95.5)
Redundancy	2.8 (2.6)
<I/σ(I)>	3.7 (0.7)
CC(1/2) (%)	99.2 (31.9)
**Refinement**	
No. of atoms	6833
Protein	6694
Water	129
Zn ions	3
Ni ions	2
Average thermal factor (Å^2^)	42.7
Wilson B-factor (Å^2^)	42.5
**r.m.s. deviations from ideality**	
Bond lengths (Å)	0.018
Bond angles	1.847
No. reflections work/free	39616/1997
R__work_/R_free__	20.8/24.2
Ramachandran plot favored/outliers (%)^†^	95.1/0.6

^†^According to the definition used in Molprobity^44^.

*Values in parenthesis refers to the outer resolution shell.

## References

[b1] Schmierer B. & Hill C. S. TGFbeta-SMAD signal transduction: molecular specificity and functional flexibility. Nat. Rev. Mol. Cell Biol. 8, 970–982 (2007). 18000526 10.1038/nrm2297

[b2] Morikawa M. . ChIP-seq reveals cell type-specific binding patterns of BMP-specific Smads and a novel binding motif. Nucleic Acids Res. 39, 8712–8727 (2011). 21764776 10.1093/nar/gkr572 PMC3203580

[b3] Baburajendran N. . Structure of Smad1 MH1/DNA complex reveals distinctive rearrangements of BMP and TGF-beta effectors. Nucleic Acids Res. 38, 3477–3488 (2010). 20147459 10.1093/nar/gkq046 PMC2879523

[b4] Baburajendran N., Jauch R., Tan C. Y. Z., Narasimhan K. & Kolatkar P. R. Structural basis for the cooperative DNA recognition by Smad4 MH1 dimers. Nucleic Acids Res. 39, 8213–8222 (2011). 21724602 10.1093/nar/gkr500 PMC3185416

[b5] Chai N. . Structural basis for the Smad5 MH1 domain to recognize different DNA sequences. Nucleic Acids Res. 43, 9051–9064 (2015). 26304548 10.1093/nar/gkv848 PMC4605309

[b6] Chan H. M. & La Thangue N. B. p300/CBP proteins: HATs for transcriptional bridges and scaffolds. J. Cell. Sci. 114, 2363–2373 (2001). 11559745 10.1242/jcs.114.13.2363

[b7] Goldman P. S., Tran V. K. & Goodman R. H. The multifunctional role of the co-activator CBP in transcriptional regulation. Recent Prog. Horm. Res. 52, 103–19– discussion 119–20 (1997). 9238849

[b8] Ng H. H. & Bird A. Histone deacetylases: silencers for hire. Trends Biochem. Sci. 25, 121–126 (2000). 10694882 10.1016/s0968-0004(00)01551-6

[b9] Hiratochi M. . The Delta intracellular domain mediates TGF-beta/Activin signaling through binding to Smads and has an important bi-directional function in the Notch-Delta signaling pathway. Nucleic Acids Res. 35, 912–922 (2007). 10.1093/nar/gkl1128 PMC1807952 17251195

[b10] Deheuninck J. & Luo K. Ski and SnoN, potent negative regulators of TGF-beta signaling. Cell Res. 19, 47–57 (2009). 19114989 10.1038/cr.2008.324 PMC3103856

[b11] Pot I. & Bonni S. SnoN in TGF-beta signaling and cancer biology. Curr. Mol. Med. 8, 319–328 (2008). 18537639 10.2174/156652408784533797

[b12] Tecalco-Cruz A. C. . Transforming growth factor-β/SMAD Target gene SKIL is negatively regulated by the transcriptional cofactor complex SNON-SMAD4. J. Biol. Chem. 287, 26764–26776 (2012). 22674574 10.1074/jbc.M112.386599 PMC3411014

[b13] Colmenares C. & Stavnezer E. Structure and activities of the ski oncogene. Semin. Cancer Biol. 1, 383–387 (1990). 2103510

[b14] Edmiston J. S., Yeudall W. A., Chung T. D. & Lebman D. A. Inability of transforming growth factor-beta to cause SnoN degradation leads to resistance to transforming growth factor-beta-induced growth arrest in esophageal cancer cells. Cancer Res. 65, 4782–4788 (2005). 15930298 10.1158/0008-5472.CAN-04-4354

[b15] Imoto I. . SNO is a probable target for gene amplification at 3q26 in squamous-cell carcinomas of the esophagus. Biochem. Biophys. Res. Commun. 286, 559–565 (2001). 11511096 10.1006/bbrc.2001.5428

[b16] Seoane J. Escaping from the TGFbeta anti-proliferative control. Carcinogenesis 27, 2148–2156 (2006). 16698802 10.1093/carcin/bgl068

[b17] Zhang L., Sato E., Amagasaki K., Nakao A. & Naganuma H. Participation of an abnormality in the transforming growth factor-beta signaling pathway in resistance of malignant glioma cells to growth inhibition induced by that factor. J. Neurosurg. 105, 119–128 (2006). 16871886 10.3171/jns.2006.105.1.119

[b18] Caligaris C. . Actin-cytoskeleton polymerization differentially controls the stability of Ski and SnoN co-repressors in normal but not in transformed hepatocytes. Biochim. Biophys. Acta 1850, 1832–1841 (2015). 26002202 10.1016/j.bbagen.2015.05.012

[b19] Roberts A. B. & Wakefield L. M. The two faces of transforming growth factor beta in carcinogenesis. Proc. Natl. Acad. Sci. USA 100, 8621–8623 (2003). 12861075 10.1073/pnas.1633291100 PMC166359

[b20] Zhu Q. . Dual role of SnoN in mammalian tumorigenesis. Mol. Cell. Biol. 27, 324–339 (2007). 17074815 10.1128/MCB.01394-06 PMC1800653

[b21] Sarker K. P., Wilson S. M. & Bonni S. SnoN is a cell type-specific mediator of transforming growth factor-beta responses. J. Biol. Chem. 280, 13037–13046 (2005). 15677458 10.1074/jbc.M409367200

[b22] Akiyoshi S. . c-Ski acts as a transcriptional co-repressor in transforming growth factor-beta signaling through interaction with smads. J. Biol. Chem. 274, 35269–35277 (1999). 10575014 10.1074/jbc.274.49.35269

[b23] Luo K. . The Ski oncoprotein interacts with the Smad proteins to repress TGFbeta signaling. Genes Dev. 13, 2196–2206 (1999). 10485843 10.1101/gad.13.17.2196 PMC316985

[b24] Nomura T. . Ski is a component of the histone deacetylase complex required for transcriptional repression by Mad and thyroid hormone receptor. Genes Dev. 13, 412–423 (1999). 10049357 10.1101/gad.13.4.412 PMC316468

[b25] Stroschein S. L., Wang W., Zhou S., Zhou Q. & Luo K. Negative feedback regulation of TGF-beta signaling by the SnoN oncoprotein. Science 286, 771–774 (1999). 10531062 10.1126/science.286.5440.771

[b26] Tokitou F. . Viral ski inhibits retinoblastoma protein (Rb)-mediated transcriptional repression in a dominant negative fashion. J. Biol. Chem. 274, 4485–4488 (1999). 9988677 10.1074/jbc.274.8.4485

[b27] Xu W. . Ski acts as a co-repressor with Smad2 and Smad3 to regulate the response to type beta transforming growth factor. Proc. Natl. Acad. Sci. USA 97, 5924–5929 (2000). 10811875 10.1073/pnas.090097797 PMC18535

[b28] Wu J. W. . Structural mechanism of Smad4 recognition by the nuclear oncoprotein Ski: insights on Ski-mediated repression of TGF-beta signaling. Cell 111, 357–367 (2002). 12419246 10.1016/s0092-8674(02)01006-1

[b29] Mizuide M. . Two short segments of Smad3 are important for specific interaction of Smad3 with c-Ski and SnoN. J. Biol. Chem. 278, 531–536 (2003). 12426322 10.1074/jbc.C200596200

[b30] Nyman T. . The crystal structure of the Dachshund domain of human SnoN reveals flexibility in the putative protein interaction surface. PLoS ONE 5, e12907 (2010). 20957027 10.1371/journal.pone.0012907 PMC2944819

[b31] He J., Tegen S. B., Krawitz A. R., Martin G. S. & Luo K. The transforming activity of Ski and SnoN is dependent on their ability to repress the activity of Smad proteins. J. Biol. Chem. 278, 30540–30547 (2003). 12764135 10.1074/jbc.M304016200

[b32] Lapkouski M. & Hällberg B. M. Structure of mitochondrial poly(A) RNA polymerase reveals the structural basis for dimerization, ATP selectivity and the SPAX4 disease phenotype. Nucleic Acids Res. 43, 9065–9075 (2015). 26319014 10.1093/nar/gkv861 PMC4605311

[b33] Acuner Ozbabacan S. E., Engin H. B., Gursoy A. & Keskin O. Transient protein-protein interactions. Protein Engineering Design and Selection 24, 635–648 (2011). 10.1093/protein/gzr025 21676899

[b34] Chacko B. M. . Structural basis of heteromeric smad protein assembly in TGF-beta signaling. Mol. Cell 15, 813–823 (2004). 15350224 10.1016/j.molcel.2004.07.016

[b35] Chacko B. M. . The L3 loop and C-terminal phosphorylation jointly define Smad protein trimerization. Nat. Struct. Biol. 8, 248–253 (2001). 11224571 10.1038/84995

[b36] Kabsch W. Evaluation of single-crystal X-ray diffraction data from a position-sensitive detector. Journal of Applied Crystallography 21, 916–924 (1988).

[b37] Evans P. Scaling and assessment of data quality. Acta Crystallogr. D Biol. Crystallogr. 62, 72–82 (2006). 16369096 10.1107/S0907444905036693

[b38] McCoy A. J. . Phaser crystallographic software. Journal of Applied Crystallography 40, 658–674 (2007). 19461840 10.1107/S0021889807021206 PMC2483472

[b39] Adams P. D. . PHENIX: a comprehensive Python-based system for macromolecular structure solution. Acta Crystallogr. D Biol. Crystallogr. 66, 213–221 (2010). 20124702 10.1107/S0907444909052925 PMC2815670

[b40] Murshudov G. N., Vagin A. A. & Dodson E. J. Refinement of macromolecular structures by the maximum-likelihood method. Acta Crystallogr. D Biol. Crystallogr. 53, 240–255 (1997). 15299926 10.1107/S0907444996012255

[b41] Emsley P. & Cowtan K. Coot: model-building tools for molecular graphics. Acta Crystallogr. D Biol. Crystallogr. 60, 2126–2132 (2004). 15572765 10.1107/S0907444904019158

[b42] Krissinel E. & Henrick K. Inference of macromolecular assemblies from crystalline state. J. Mol. Biol. 372, 774–797 (2007). 17681537 10.1016/j.jmb.2007.05.022

[b43] Ashkenazy H., Erez E., Martz E., Pupko T. & Ben-Tal N. ConSurf 2010: calculating evolutionary conservation in sequence and structure of proteins and nucleic acids. Nucleic Acids Res. 38, W529–33 (2010). 20478830 10.1093/nar/gkq399 PMC2896094

[b44] Davis I. W. . MolProbity: all-atom contacts and structure validation for proteins and nucleic acids. Nucleic Acids Res. 35, W375–83 (2007). 17452350 10.1093/nar/gkm216 PMC1933162

