# Supplementary material for: SnoN Stabilizes the SMAD3/SMAD4 Protein Complex
Source: Sci Rep. 2017 Apr 11;7:46370. doi: 10.1038/srep46370 (PMC5387736; doi:10.1038/srep46370)
Supplement: Supplementary Information [file srep46370-s1.pdf]

SUPPLEMENTARY INFORMATION

SnoN Stabilizes the SMAD3/SMAD4 Protein Complex

Karin Walldén [†], Tomas Nyman [‡] and B. Martin Hällberg [†, §, ||, *]

[†] Department of Cell and Molecular Biology, Karolinska Institutet, 171 77 Stockholm, Sweden

[‡] Department of Medical Biochemistry and Biophysics, Karolinska Institutet, 171 77 Stockholm, Sweden

[§] Röntgen-Ångström-Cluster, Karolinska Institutet Outstation, Centre for Structural Systems Biology, Centre for Structural Systems Biology, DESY-Campus, 22603 Hamburg, Germany

[||] European Molecular Biology Laboratory, Hamburg Unit, 22603 Hamburg, Germany

* Correspondence and requests for material should be addressed to: B.M.H. (email: Martin.Hallberg@ki.se)

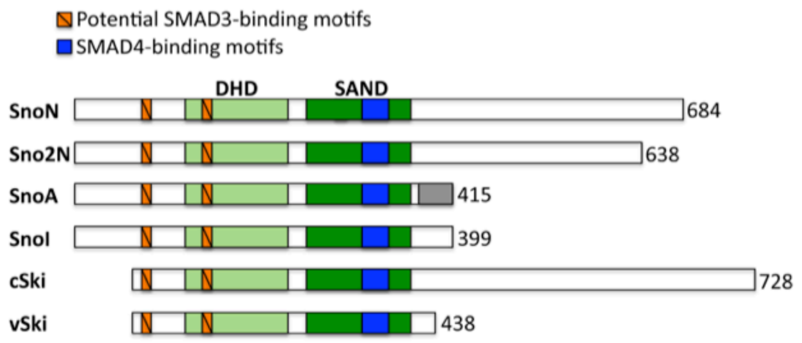

Figure S1. Domain architecture of SnoN (human) and Ski (human cSki and chicken vSki) isoforms.

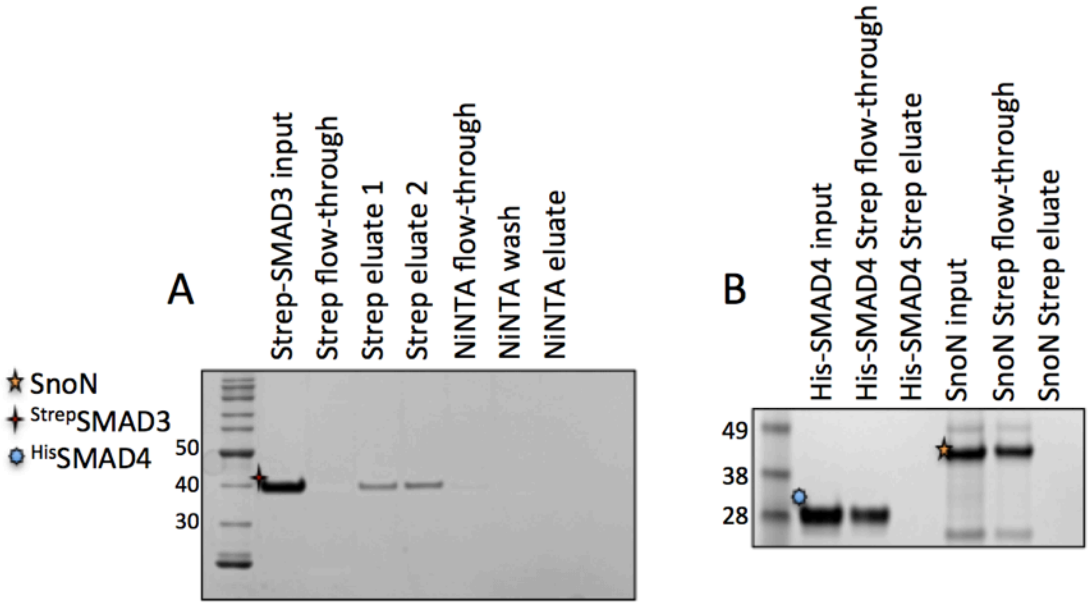

Figure S2. Pull-down controls.
StrepSMAD3 does not bind unspecifically to NiNTA resin at 50 mM imidazol; HisSMAD4 does not bind unspecifically to streptactin resin; and SnoN does not bind unspecifically to Streptactin resin. Separate affinity purifications were performed of SMAD3 (33.6 kDa), SMAD4 (28.3 kDa) and SnoN (38.6 kDa), respectively. Fractions were analyzed with 4-12% Bis-Tris SDS-PAGE gel electrophoresis followed by Coomassie staining. (**A**) From left, StrepSMAD3 input; sample not sticking to Strep-Tactin column (Strep flow-through); Strep-Tactin eluate fractions 1 and 2 (Strep eluate 1, Strep eluate 2); Strep-Tactin elute was loaded directly onto NiNTA column, and any proteins not sticking to the NiNTA column (NiNTA flow-through and NiNTA wash); and NiNTA eluate. (**B**) From left, HisSMAD4 input; HisSMAD4 sample not sticking to Strep-Tactin column (Strep flow-through); HisSMAD4 Strep-Tactin eluate; SnoN input; SnoN sample not sticking to Strep-Tactin resin (Strep flow-through); SnoN Strep-Tactin eluate. BenchMark Unstained Protein Ladder (Invitrogen) and SeeBlue® Plus2 Pre-stained Protein Standard (Invitrogen) were used for A and B, respectively, with relevant molecular weights indicated to the left of each gel.

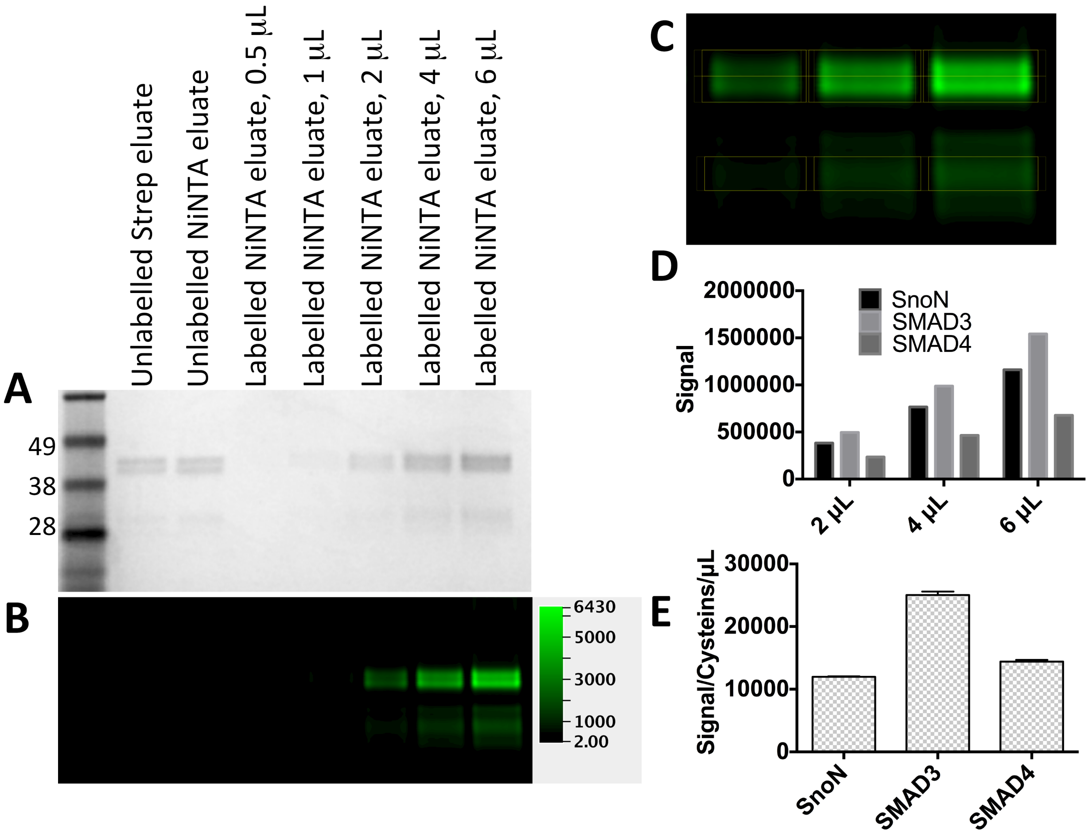

Figure S3. Stoichiometry of SnoN-SMAD3-SMAD4 complex.

To assess the stoichiometry of the SnoN-SMAD3-SMAD4 complex (purified as shown in Figure 1A), cysteins were labelled with IRDye 800CW (LI-COR Biotechnology GmbH)and fluorescence was measured at 800 nm. Volume loaded in each lane is indicated for each lane. (**A**) Coomassie stained gel. SeeBlue® Plus2 Pre-Stained Standard (Invitrogen) was used. (**B**) Gel scanned at 800 nM using Odyssey Infrared Imaging System (LI-COR Biotechnology GmbH). Color/signal bar is shown to the left. (**C**) Analysis of bands corresponding to 2 µL, 4 µL and 6 µL sample was performed using Image Studio Lite (LI-COR Biotechnology GmbH), with the areas used for analysis indicated by the shown boxes. (**D**) Signals plotted for the lanes corresponding to 2 µL, 4 µL and 6 µL sample. (**E**) Signal divided with number of cysteines (16, 10 and 8 for SnoN, SMAD3 and SMAD4, respectively) and amount sample loaded is plotted for each protein. This corrected signal is now linear to the molar amount of each protein in the lane. From this the stoichiometry was calculated to 1.0:2.1:1.2 (SnoN:SMAD3:SMAD4), as indicated in Figure 1E. Standard deviations are indicated with error bars. Plots were made using GraphPad Prism version 6.

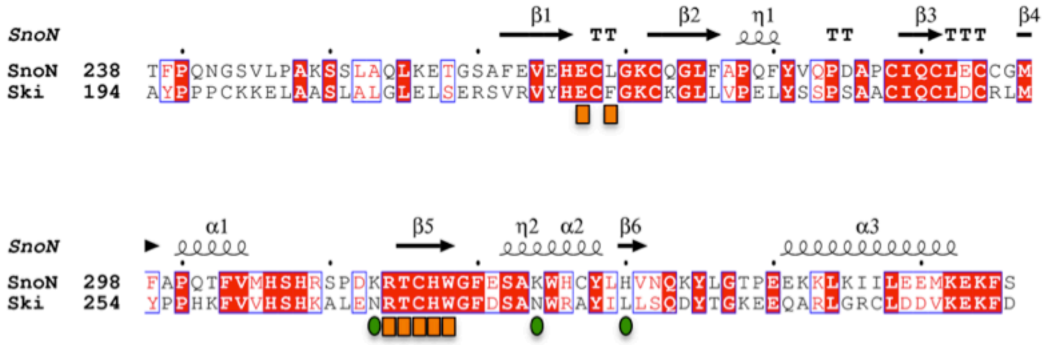

Figure S4. SnoN-SMAD4 and R-SMAD/SMAD4 comparison.
Sequence alignment of SAND domains of SnoN and Ski. Residues of SnoN involved in electrostatic interactions are indicated in orange (found in both open and closed form) and green (found only in closed form). Secondary structure elements are indicated for SnoN.

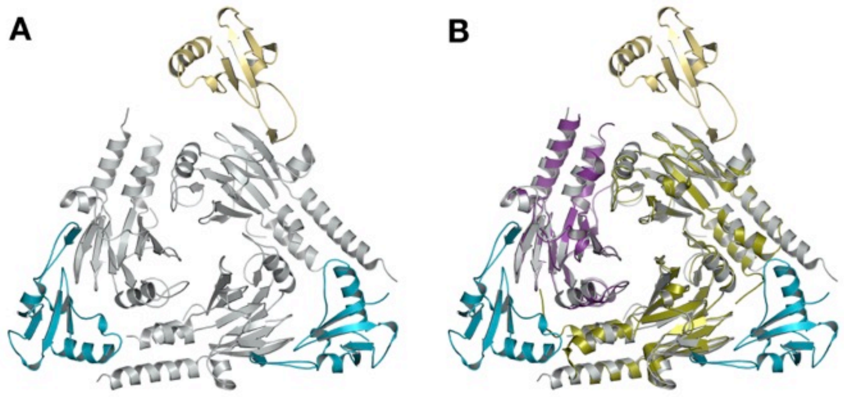

Figure S5. SnoN-SMAD4 and R-SMAD/SMAD4 comparison.
The complex of SnoN and SMAD4 assembles in the crystallographic asymmetric unit as trimers of heterodimers, and SMAD4 forms similar trimer contacts as R-SMAD/SMAD4. (**A**) Assymetric unit content of SnoN-SMAD4. SnoN is present both in the open (beige) and closed (cyan) conformation. SMAD4 (grey) conformation is unchanged within the heterohexameric assembly. (**B**) SnoN-SMAD4 structure is superimposed on the structure of SMAD3 (lilac)/SMAD4 (green-yellow) complex (pdb code 1U7F).

Figure S6. Mass Spectrometry analysis.
MALDI-MS/MS mass spectrometry analysis of trypsinated SMAD4 protein extracted from SMAD4 band of double pulldown of SnoN, SMAD3 and SMAD4. In red are identified peptides.

>SMAD4 MH2
MHHHHHHSSGVDLGTENLYFQSISNHPAPEYWCSIAYFEMDVQVGETFKVPSSCPIVTVDGYVD
PSGGDRFCLGQLSNVHRTEAIERARLHIGKGVQLECKGEGDVWVRCLSDHAVFVQSYYLDREAG
RAPGDAVHKIYPSAYIKVFDLRQCHRQMQQQAATAQAAAAAQAAAVAGNIPGPGSVGGIAPAIS
LSAAAGIGVDDLRRLCILRMSFVKGWGPDYPRQSIKETPCWIEIHLHRALQLLDEVLHTMPIADPQ
